# The effect of seasonal heat acclimatization on cool‐seeking behaviour during passive heat stress in young adults

**DOI:** 10.1113/EP091969

**Published:** 2024-09-09

**Authors:** Hui Wang, Zachary J. Schlader, Tze‐Huan Lei, Toby Mündel, Tatsuro Amano, Naoto Fujii, Takeshi Nishiyasu, James Cotter, Narihiko Kondo

**Affiliations:** ^1^ Laboratory for Applied Human Physiology, Graduate School of Human Development and Environment Kobe University Kobe Japan; ^2^ Department of Kinesiology Indiana University School of Public Health Bloomington Indiana USA; ^3^ College of Physical Education Hubei Normal University Huangshi China; ^4^ Department of Kinesiology Brock University St. Catharines ON Canada; ^5^ Faculty of Education Niigata University Niigata Japan; ^6^ Institute of Health and Sports Science University of Tsukuba Tsukuba Japan; ^7^ School of Physical Education, Sport and Exercise Sciences University of Otago Dunedin New Zealand

**Keywords:** behavioural thermoregulation, electric fan, passive heat stress, seasonal heat acclimatization

## Abstract

Seasonal heat acclimatization is known to enhance autonomic thermoeffector responses, whereas the behavioural response following seasonal heat acclimatization remains unknown. We investigated whether seasonal heat acclimatization would alter autonomic and behavioural thermoregulatory responses. Sixteen healthy participants (eight males and eight females) underwent two trials involving 50 min of lower‐leg passive heating (lower‐leg submersion in 42°C water) with (Fan trial) and without (No fan trial) the voluntary use of a fan in a moderate thermal environment (27°C, 50% relative humidity) across winter and summer months. In Fan trials, participants were allowed to use a fan to maintain thermal comfort, but this was not allowed in the No fan trials. Cool‐seeking behaviour was initiated at a lower change in rectal temperature [mean (SD): 0.21 (0.18)°C vs. 0.11 (0.13)°C, *P *= 0.0327] and change in mean skin temperature [2.34 (0.56)°C vs. 1.81 (0.32)°C, *P <* 0.0001], and cooling time was longer [16.46 (5.62) vs. 20.40 (4.87) min, *P =* 0.0224] in summer compared with winter. However, thermal perception was not modified by season during lower‐leg passive heating (all *P >* 0.0864). Furthermore, rectal temperature was higher in summer (*P =* 0.0433), whereas mean body temperature and skin temperature were not different (all *P >* 0.0631) between the two seasons in Fan trials. In conclusion, seasonal heat acclimatization enhanced the cool‐seeking behaviour from winter to summer.

## Abstract (highlights)

- **What is the central question of this study?**
  Repeated heat exposure during the summer months (seasonal heat acclimatization) can enhance autonomic thermoregulatory responses in humans, but does it also modify cool‐seeking behaviour during passive heat exposure?
- **What is the main finding and its importance?**
  Although seasonal heat acclimatization enhanced cool‐seeking behaviour in young adults during lower‐leg passive heating, it was initiated earlier, more frequently in the summer compared with the winter. These adaptations indicate that thermal behaviour might complement the autonomic thermoregulatory response.

## 1 INTRODUCTION

During acute heat exposure, the activation of behavioural and autonomic thermoeffectors is essential to maintain thermal homeostasis (Schlader & Vargas, 2019). It is well established that heat dissipation from autonomic thermoeffector activation is increased with seasonal heat acclimatization or heat acclimation owing to repeated exposure to heat stress. The typical autonomic thermoregulatory adaptations following seasonal heat acclimatization include a higher local and whole‐body sweating rate, a lower sweat ion concentration and a smaller rise in core temperature during passive heating and exercise (Banjar et al., 1989; Inoue et al., 1995; Lee et al., 2015; Lei et al., 2021; Lorenzo & Minson, 2010; Nakamura & Okamura, 1998).

Like the autonomic thermoregulatory adaptations following seasonal heat acclimatization, it is likely that behavioural thermoregulatory responses would be altered. Previous studies observed that rats housed in high temperatures groom and spread saliva (i.e., enhance evaporative heat loss) more often and have lower spontaneous locomotor activity (i.e., reduce metabolic heat production) during long‐term heat exposure (Horowitz et al., 1983; Michel et al., 2010; Nakagawa et al., 2020). However, how human behaviour and perceptual responses are adapted to heat stress with seasonal heat acclimatization remains unknown.

Cool‐seeking behaviours are initiated by the sensation of thermal discomfort caused by elevations in body temperature (Bulcao et al., 2000; Schlader et al., 2011) and skin wetness (Vargas et al., 2020). Lee et al. (2011) identified that tropical indigenes were less perceptually sensitive to warm and hot stimuli than temperate indigenes at rest. Another study investigated whether heat acclimation modifies the thermal comfort zone by using a water‐perfused suit and reported that most participants felt warmer yet more comfortable during whole‐body hot water immersion after heat acclimation, in comparison to before heat acclimation (Sotiridis et al., 2020). Given that mean skin temperature and autonomic thermoeffector activity (e.g., eccrine sweating) are usually higher following seasonal heat acclimatization (Banjar et al., 1989; Inoue et al., 1995; Lei et al., 2021; Nakamura & Okamura, 1998), it is reasonable to speculate that cool‐seeking behaviour is modified by seasonal heat acclimatization. Thus, based on these studies, it is likely that seasonal heat acclimatization modifies cool‐seeking behaviour during passive heat stress.

The electric fan is a cost‐effective and easily accessible personal cooling modality that is widely used in daily life (Jay et al., 2015, 2019) and enhances whole‐body evaporation during passive heating and exercise (Gupta et al., 2012; Ravanelli et al., 2015). We have shown that voluntarily turning on a fan during passive heating affects autonomic thermoeffectors and perceptual responses (Wang et al., 2023). However, the effect of seasonal heat acclimatization on the initiation of cool‐seeking behaviour in a hot, humid climate (e.g., Japan) remains unknown. Therefore, the aim of this study was to investigate the effect of seasonal heat acclimatization between the winter and summer months on cool‐seeking behaviour during passive heat stress. We tested the hypothesis that cool‐seeking behaviour during passive heat stress will be initiated earlier and more frequently in the summer compared with winter.

## 2 MATERIALS AND METHODS

The Human Subjects Committee of the Graduate School of Human Development and Environment at Kobe University (Japan) approved the study (No. 425), which conforms to the standards set out by the latest version of the *Declaration of Helsinki*. Participants were informed about the purpose and procedures of the study prior to providing verbal and written consent.

### 2.1 Participants

Based on prior research (Hunt et al., 2021) and pilot studies, we estimated an effect size (Cohen's *d*) of 0.7. Using this effect size, α = 0.05 and 1 − *β* = 0.80, a minimum sample size of 15 participants is required to *t*‐test (G*Power (Power analysis is essential for determining the sample size required to detect an effect of a given size with a particular level of confidence (statistical power))). Therefore, 16 healthy young adults without a history of exercise training (eight females) were recruited for this study; a subset participants (including four males and five females) who completed 50 min of lower‐leg passive heating in the winter months as part of another investigation (Wang et al., 2023) were re‐evaluated using the same heat stress test during the following summer. The physical characteristics of participants are summarized in Table 1. All participants were familiar with the study equipment and approach, without knowing the research hypotheses and did not take any medications. Participants did not report any known neurological, metabolic, cardiovascular or mental illnesses. All females were on contraception and self‐reported to be menstruating regularly and were tested within the early/mid‐follicular phase, defined as the first 10 days from the onset of menstruation, to control for the influence of female sex hormones (Vargas et al., 2020).

**TABLE 1:** The physical characteristics across both winter and summer seasons (*N* = 16).

Season	Age (year)	Height (cm)	Mass (kg)	Bodyfat (%)	${\dot{V}}_{O_{2}\mathrm{peak}}$ (mL/kg/min)
Winter	24.3 (3.6)	165.9 (8.8)	57.9 (7.8)	22 (7.4)	38.2 (8.8)
Summer	24.4 (4.1)	165.9 (9.6)	56.3 (9.5)	21 (7.9)	33.0 (8.0)
*P*‐value	0.898	0.865	0.583	0.118	0.0279^*^

Abbreviation: ${\dot{V}}_{O_{2}\mathrm{peak}}$, peak oxygen uptake.

^*^Significant difference between winter and summer seasons (*P*‐values are reported).

### 2.2 Experimental overview

In each season (winter and summer), participants were asked to visit the laboratory on three occasions. Visit 1 involved pilocarpine iontophoresis and maximal oxygen consumption (${\dot{V}}_{O_{2}\mathrm{peak}}$) tests, and visits 2 and 3 involved lower‐leg passive heating without and with an electric fan. Each visit was separated by ≥48 h and was conducted in the morning between 08.00 and 11.00 h, to minimize the effect of circadian rhythms, and 2 h postprandial. All visits were performed in a fixed order, with the electric fan omitted during the visit 2 and included in the visit 3. This was chosen, instead of a random order, to ensure that everyone had the same previous experience with the passive heating protocol. However, the winter session and summer session were conducted in a random order. All trials were conducted between the middle of January and the end of February (winter) and between the middle of July and the end of August (summer) in Kobe, Japan. The average ambient conditions in summer and winter are described in Table 2 (data from the Japan Meteorological Agency).

**TABLE 2:** The ambient conditions in summer and winter (data from the Japan Meteorological Agency).

	Ambient temperature (°C)			Humidity	
Season	Average	Lowest	Highest	Average RH (%)	Average absolute humidity (g/m^3^)
Winter	6.2	3.1	10.1	61	4
Summer	28.6	24.7	32.2	74	19

Abbreviation: RH, relative humidity.

### 2.3 Lower‐leg passive heating

The passive heat stress protocol began with submerging the legs up to the knees in a 42°C stirred water bath (Crandall & Wilson, 2015; Heinonen et al., 2011; Inoue et al., 2005; Kuwahara, Inoue, Abe et al., 2005; Kuwahara, Inoue, Taniguchi et al., 2005) while the participants were in a semi‐supine position. This moderate level of heat exposure allowed for gradual elevation of core temperature, thus enabling the evaluation of both changes in the time courses of autonomic thermoeffectors, and the responsiveness of cool‐seeking behavior with increasing body temperatures. Moreover, this approach has less central fluid displacement and more realistic mean skin temperatures in the non‐immersed areas compared with whole‐body immersion or heating with a water‐perfused suit (Crandall & Wilson, 2015). Additionally, it has been reported that changes in core temperature contribute strongly to the initiation of thermoregulatory behaviour in hyperthermic conditions (Schlader & Vargas, 2019), and compared with heat stress in a climate chamber, the lower‐leg passive heating allowed more internal heat strain than external (i.e., cutaneous) heat strain.

### 2.4 Pilocarpine iontophoresis and measurement of ${\dot{V}}_{O_{2}\mathrm{peak}}$

We used the pilocarpine‐induced sweating test to evaluate the degree of seasonal heat acclimatization, because local sweat adaptation is one of the key markers for heat acclimatization (Gerrett et al., 2021). Also, because ${\dot{V}}_{O_{2}\mathrm{peak}}$, as an indicator of aerobic fitness is likely to affect autonomic thermoeffector responses (Kuwahara Inoue, Taniguchi et al., 2005), we measured ${\dot{V}}_{O_{2}\mathrm{peak}}$ in winter and summer.

The pilocarpine‐induced sweating test was performed using the iontophoresis method as an indication of cholinergic sweat gland function (Webster & Rundell, 1982) and as an index of seasonal heat acclimatization from winter to summer (Shin et al., 2016). Before data collection, participants were seated quietly for 30 min in an environmental chamber (model FLC 2700s; Fuji Medical Science, Japan) at an ambient temperature of 25°C and relative humidity (RH) of 50%. During this period, 1% (equivalent to 0.05 m) pilocarpine (Tokyo Chemical Industry Co., Ltd, Japan) was applied iontophoretically onto the distal ventral forearm via a capsule (5.31 cm^2^) dissolved in distilled water via gauze. Then a 1.5 mA iontophoresis current was applied for 5 min between an electrode on the pilocarpine capsule and a flexible plate electrode (cathode, HV‐LLPD; Omron Healthcare, Japan) attached to the distal ventral forearm. Immediately after iontophoresis, the iontophoresis capsule was removed, the skin surface was wiped with clean gauze, and another sweat capsule (5.31 cm^2^) was attached at the same location for measurement of sweat rate using the ventilated capsule method with an airflow of 0.6 L/min. Although this procedure took 12 min overall, only the final 10 min were averaged for data analysis. After the test, the capsule was removed, and an active sweat gland test was conducted, using the starch–iodine technique described by Kenney and Fowler (1988). The ratio between the average forearm sweat rate and the active sweat gland was used to calculate sweat gland output of the forearm.

After the active sweat gland measurement, each participant underwent a ${\dot{V}}_{O_{2}\mathrm{peak}}$ test by cycle ergometer (Aerobike, 75XLIII; Konami, Japan), with a workload that began at 20 W for the first 2 min and increased by 15 W every minute for females or 30 W every minute for males, and maintained a cadence of 60 rpm until volitional exhaustion. The criteria for ${\dot{V}}_{O_{2}\mathrm{peak}}$ were if three of the following five conditions were met: a respiratory exchange ratio ≥1.10; a plateau in O_2_ uptake with increasing workloads; workload volitional fatigue (<55 rpm cadence); exercise heart rate within 10 beats of the age‐predicted maximal heart rate [(220 − age) × 0.95]; and rating of perceived exertion (RPE) of 19–20 (Rivas et al., 2020).

### 2.5 Experimental trials

**FIGURE 1:**
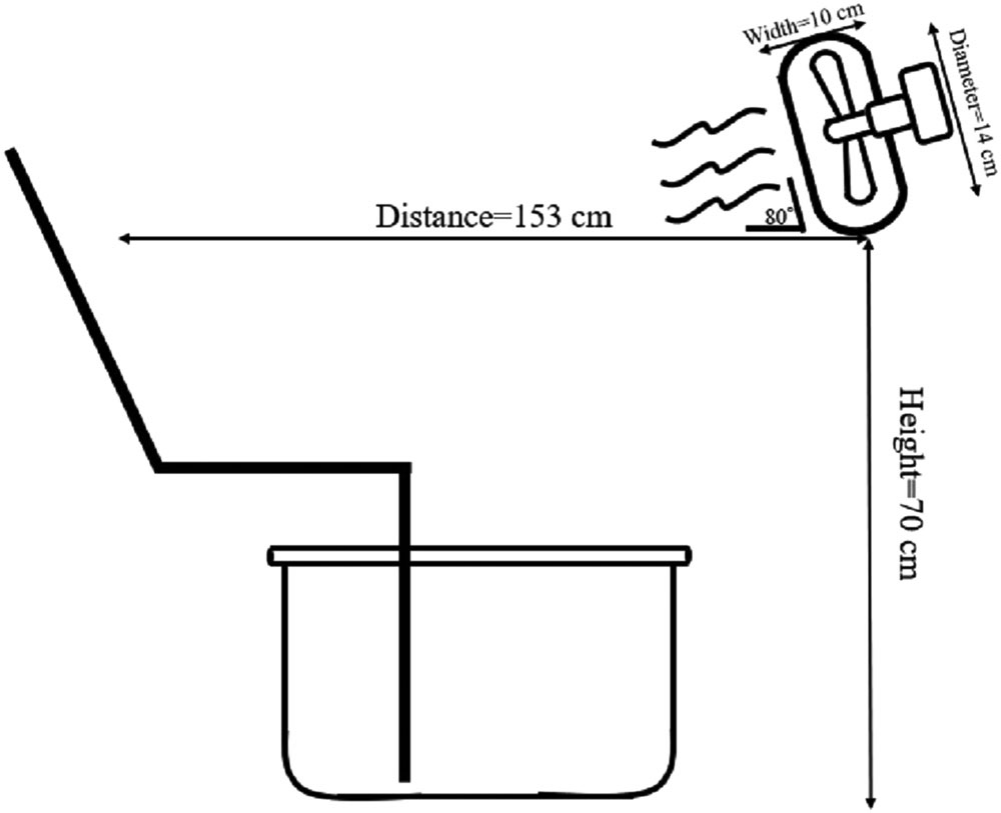
Schematic diagram for the position of the fan.

Before entering the chamber, participants provided a urine sample for the assessment of hydration status. Euhydration was confirmed by urine‐specific gravity, with no participants exceeding the value of 1.015 (Cheuvront & Sawka, 2005). Nude body mass was also measured using high‐precision weighing scales, and a rectal thermometer was then self‐inserted 10–12 cm beyond the anal sphincter. All participants in the experimental trials wore shorts (and a sports bra for females) in a climatic chamber (maintained at 27°C and 50% RH). The climatic chamber maintains temperature at ±0.3°C and RH at ±3%. Participants were asked to rest in the chamber for 50 min to get used to the ambient temperature before starting each trial. Each trial consisted of a 5 min baseline, a 50 min lower‐leg immersion and a 30 min skin blood flow (SkBF) maximum test. Participants were not allowed to talk or drink in any trials. Figure 1 depicts the experimental set‐up involving the fan placement and lower‐leg hot water immersion while in the semi‐supine position. In the Fan trial, participants were allowed to thermoregulate behaviourally by pressing a button to turn on the fan as often as they desired, with the only instruction to maintain thermal comfort. Upon pressing the button, the fan would turn on for 2 min, and there was a mandatory 1 min washout phase, which ensured a continual drive to seek cooling (Vargas et al., 2020). A special switch was custom‐made for controlling the fan (MaP3058ASCD; Nihon Santec, Osaka, Japan). In the No fan trial, participants were not allowed to turn on the fan. Thermal perceptions were measured at 10 min intervals in both trials. The average airflow of the fan was not different between winter and summer [mean (SD); 0.71 (0.07) vs. 0.72 (0.06) m/s, *P =* 0.98; Figure 1].

### 2.6 Measurements

#### 2.6.1 Physical characteristics

The height and mass were measured using a stadiometer (Seca, Germany; accurate to 0.1 cm) and scale (Mettler‐Toledo, Germany; accurate to 10 g). The percentage of body fat was determined by INNER SKIN, which used bioelectrical impedance analysis (4C Technology, TANITA Corporation, Japan).

#### 2.6.2 Airflow

The airflow of the fan was measured by an anemometer (KANOMAX, Japan). The mean value was calculated by the airflow of four body sites (forearm, abdomen, chest and neck).

#### 2.6.3 Cardiovascular parameters

Heart rate (HR) was recorded from the detection of R–R intervals (Polar Vantage XI; Polar Electro, Finland), and blood pressure was measured continuously using a Finometer device (Finapres Medical Systems, Amsterdam, The Netherlands) throughout the experimental sessions.

Skin blood flow was measured on the left mid‐medial forearm (SkBF_forearm_) and left upper chest (SkBF_chest_) using laser‐Doppler velocimetry (ALF21; Advanced, Tokyo, Japan). A 3.8‐cm‐diameter hollow circle heater element with an integrated laser Doppler flow probe inside the hollow circle was used to standardize maximal SkBF (SkBF_max_). To induce local heating‐induced maximal cutaneous vasodilatation, local skin temperature was increased to 42°C (Cui et al., 2006). Unfortunately, blood pressure could not be measured during local heating. Thus, we reported SkBF as an index of cutaneous vasomotor activity, given that blood pressure did not differ throughout the two conditions, which was also used in our previous study (with 12 participants) (Lei et al., 2021). We excluded one male subject because we failed to measure the SkBF_max_ on the chest. All SkBF values during lower‐leg passive heating were converted to percentages of the SkBFmax value [%max = (SkBF/SkBF_max_) × 100].

#### 2.6.4 Core and skin temperatures

Rectal temperature (*T*_re_) and local skin temperature on the thigh (*T*_thigh_), calf (*T*_calf_), chest (*T*_chest_) and forearm (*T*_forearm_) were measured continuously using copper–constantan thermocouples (Inui Engineering, Higashi Osaka, Japan) and secured using surgical tape (3M Micropore). To measure *T*_re_, a rectal thermocouple (Physitemp, RET‐1, USA) was inserted 10−12 cm beyond the anal sphincter. Mean skin temperature (*T*_sk_) was calculated according to the equation of Ramanathan (1964). The *T*_re_ and local skin temperature were recorded by a data logger with a sampling rate of 1 Hz and displayed on the monitor continuously. Data were expressed as a 5 min average until the end of the trial. In addition, mean body temperature (*T*_b_) was calculated as 0.8*T*_re_ + 0.2*T*_sk_ (Hardy & Stolwijk, 1966).

#### 2.6.5 Sudomotor function measurements

The sweat capsule technique was used to measure the local sweat rate (LSR) on the left mid‐medial forearm (LSR_forearm_) and left upper chest (LSR_chest_). At least 30 min before data collection, collodion glue was used to attach each capsule (3.46 cm^2^) to the skin, and the capsules were ventilated with dry air at 0.5 L/min. The effluent gas was sensed for humidity and temperature (Vaisala, Finland) and was recorded continuously by MX100 software (Yokogawa, Japan). Using high‐precision weighing scales (B60; Mettler‐Toledo, Germany), the nude body mass of the participant was measured before and after lower‐leg passive heating for the assessment of whole‐body sweat rate (WBSR).

#### 2.6.6 Behavioural thermoregulation

The primary thermoregulatory behavioural variables were the time when the first behaviour occurred (i.e., first time the fan was turned on voluntarily), the cumulative time the fan was turned on, the cumulative number of times the button was pressed, the total cooling time (i.e., the overall amount of time that the fan was turned on) and the slope of the cumulative times of pressing the button plotted ∆*T*_b_, which offered quantitative measurements of cool‐seeking behaviour (Vargas et al., 2020). Furthermore, the *T*_re_, *T*_sk_, *T*_b_ and the magnitude of increase from baseline to first behaviour initiated were also analysed.

#### 2.6.7 Thermal perceptions

Whole‐body thermal perceptions used the following standard scales: thermal sensation (−3 = very cold, −2 = cold, −1 = cool, 0 = neutral, +1 = warm, +2 = hot, +3 = very hot); thermal discomfort (−3 = very uncomfortable, −2 = uncomfortable, −1 = slightly uncomfortable, 0 = neutral, +1 = slightly comfortable, +2 = comfortable, +3 = very comfortable) and perceived wetness (−3 = very wet, −2 = wet, −1 = slightly wet, 0 = neutral, +1 = slightly dry, +2 = dry, +3 = very dry) (Olesen & Brager, 2004).

### 2.7 Data and statistical analyses

To determine the *T*_b_ at onset of LSR and SkBF in the forearm and chest region, we employed 1 min averages. The LSR and SkBF were plotted against the change in *T*_b_ from baseline (∆*T*_b_) during lower‐leg passive heating. The ∆*T*_b_ at onset for sweating and cutaneous vasodilatation were analysed using segmental regression according to Cheuvront et al. (2009). The slope of the regression line between the point of onset and before the plateau was used to determine the thermal slope of the cutaneous vasodilatation or sweating response. We also evaluated the slope of the cumulative times of pressing the button for fan use plotted against *T*_b_ as the responsiveness of cool‐seeking behaviour to increases in body temperature; this slope is indicative of how much a participant desired behaviour after initiating it.

Consistent with our hypothesis, the effect of seasonal heat acclimatization on cool‐seeking behaviour during passive heat stress was examined using Student's paired *t*‐test for behavioural variables (Table 3) and a two‐way repeated‐measures ANOVA (season × time) for the rest of the parameters in the Fan trials only. To understand the natural physiological responses to passive heating following seasonal acclimatization and ensure that any observed effects in the ‘Fan’ condition could be attributed specifically to the use of the fan, we also examined the effect of seasonal acclimatization on perceptual and autonomic responses during passive heat stress during a situation in which cool‐seeking behaviour was analysed using a two‐way repeated‐measures ANOVA (season ×  time) in the No fan trials (control condition) only. By design, however, comparisons between Fan and No fan trials were not conducted, given that the physiological effect of voluntary fan use in similar circumstances has been reported previously (Wang et al., 2023). When an ANOVA revealed a statistically significant interaction, *post hoc* comparisons were made using Sidak adjusted comparisons. Resting autonomic and cardiovascular data between seasons were analysed by Student's paired *t*‐test. The statistically significant difference was set at *P < *0.05. Descriptive statistics were presented as the mean (SD), and all variables were reported as the mean ± SD and 95% confidence intervals. The normality of the data was examined by the Kolmogorov–Smirnov test. All data were processed by GraphPad Prism software (Prism, v.8; San Diego, USA). The complete set of raw data is available in the Supporting Information.

**TABLE 3:** The behavioural thermoregulatory assessment of electric fan use across winter and summer sessions (*n* = 16).

Season	First turning on the fan (min)	Cumulative times of turning on fan (*n*)	Total cooling time (min)	∆*T*_re_ when first turning on the fan (°C)	∆*T*_sk_ when first turning on the fan (°C)	∆*T*_b_ when first turning on the fan	*T*_re_ when first turning on the fan (°C)	*T*_sk_ when first turning on the fan (°C)	*T*_b_ when first turning on the fan
Winter	17.8 (7.2)	8.7 (3.0)	16.5 (5.6)	0.2 (0.2)	2.3 (0.5)	0.6 (0.2)	37.0 (0.3)	35.1 (0.5)	36.7 (0.3)
Summer	13.3 (4.9)	10.3 (2.9)	20.4 (4.9)	0.1 (0.1)	1.8 (0.3)	0.4 (0.1)	37.1 (0.2)	35.4 (0.4)	36.8 (0.3)
*P*‐value	0.0248^*^	0.0413^*^	0.0224^*^	0.0327^*^	<0.0001^*^	<0.0001^*^	0.147	0.943	0.112
Effect size, *d*	0.64	0.60	0.63	0.59	0.88	1.37	0.33	0.02	0.42

Abbreviations: ∆, change; *T*_b_, mean body temperature; *T*_re_, rectal temperature; *T*_sk_, skin temperature.

^*^Significant difference between winter and summer seasons (*P*‐values are reported).

## 3 RESULTS

### 3.1 Physical characteristics

Following seasonal heat acclimatization, participants had a lower ${\dot{V}}_{O_{2}\mathrm{peak}}$ (*P =* 0.0279, Table 1) in summer compared with winter, whereas age, height, mass and the percentage of body fat were similar between seasons (all *P >* 0.118).

### 3.2 Pilocarpine‐induced sweat rate

Pilocarpine‐induced sweat rate was higher in summer than in winter [0.69 (0.26) vs. 0.60 (0.21) mg/cm^2^/min, *P =* 0.00132] with also higher active sweat gland (ASG) in summer than in winter [122 (32) vs. 137 (35) glands/cm^2^, *P =* 0.00433, *d *= 0.82]. However, the sweat gland output (SGO) was not different between the two seasons [5.01 (1.89) vs. 4.88 [1.54] µg/gland/min, *P = *0.492]. The average forearm skin temperature in the pilocarpine‐induced sweating test was significantly higher in summer than in winter [32.9 (0.5)°C vs. 31.2 (0.7)°C, *P <* 0.0001].

### 3.3 Effect of seasonal heat acclimatization on cool‐seeking behaviour during passive heat stress

#### 3.3.1 Cool‐seeking behaviour parameters

**FIGURE 2:**
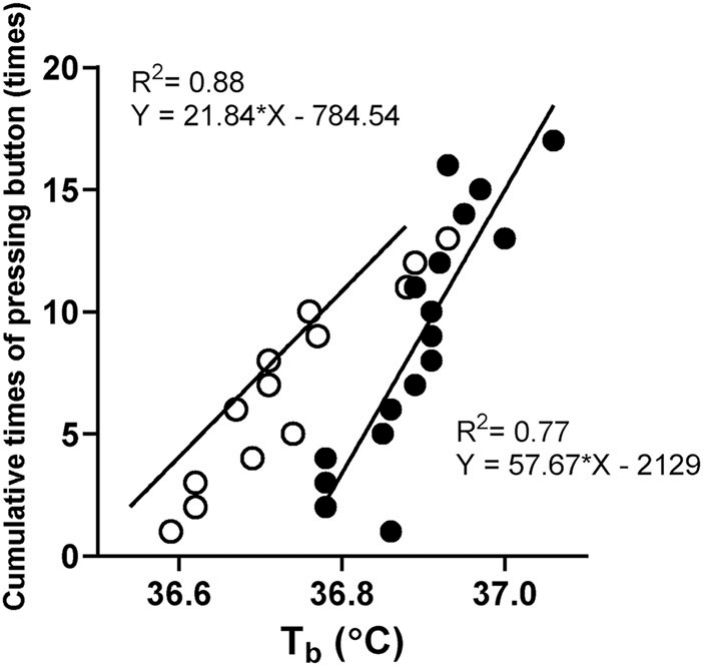
Relationship between the cumulative number of button presses and change in mean body temperature (∆*T*_b_) during passive heating in a participant (*n* = 1), which depicts how much thermal behaviour was desired (slope) in winter trials (open circles) and summer trials (filled circles).

The onset of fan use was earlier in summer compared with winter (*P =* 0.0224; Table 3). Likewise, total cooling time and cumulative time of pressing the button were higher in summer compared with winter (all *P <* 0.0413; Table 3). Moreover, the fan was initiated at a lower *∆T*_re_, *∆T*_sk_ and *∆T*_b_ in summer (all *P <* 0.0327; Table 3), although not for absolute *T*_re_, *T*_sk_, and *T*_b_ (all *P >* 0.112; Table 3). Figure 2 shows the relationship between cumulative times of pressing the button and rising *T*_b_ in a representative participant. The slope of the relationship was significantly higher in summer than in winter [21.84 (12.08) vs. 29.92 (17.53) button presses/°C, *P =* 0.0327, *d* = 0.83].

Furthermore, cool‐seeking behaviour was initiated at a lower ∆*T*_b_ compared with the onset of SkBF and LSR in both winter and summer [onset of SkBF: 0.64 (0.07)°C in winter, 0.50 (0.11)°C in summer; onset of LSR: 0.63 (0.15)°C in winter, 0.54 (0.07)°C in summer, all *P <* 0.0436].

#### 3.3.2 Body temperature

**FIGURE 3:**
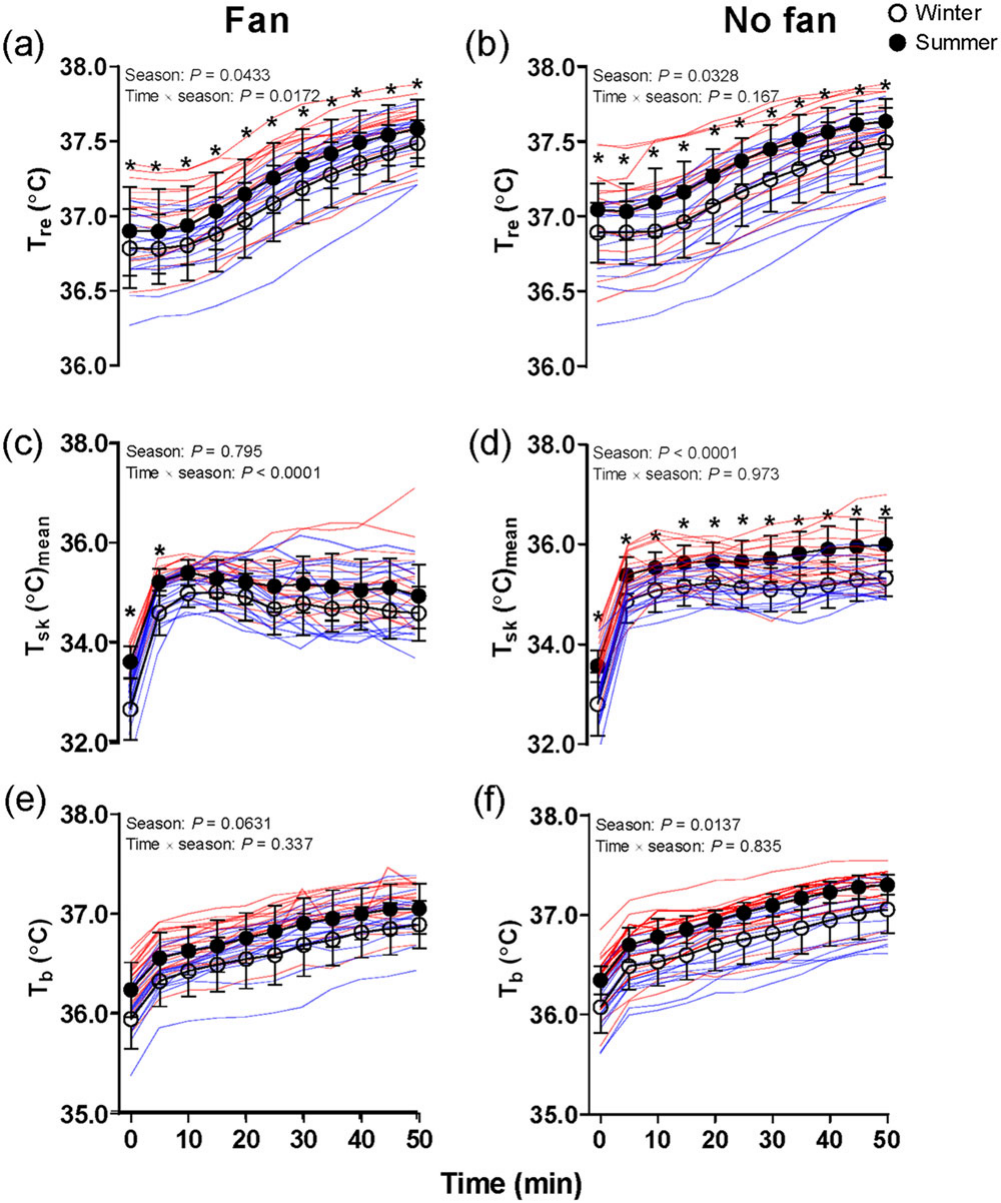
Time courses of changes (∆) in rectal temperature (*T*_re_; a, b), mean skin temperature (*T*_sk_; c, d) and mean body temperature (*T*_b_; e, f) during lower‐leg immersion between winter and summer in Fan and No fan trials. *n* = 16 (8 male and 8 female). Filled circles are group means (SD) in summer, and open circles indicate group means (SD) in winter. The blue lines are individual responses in winter, and the red lines indicate the individual responses in summer. ^*^Significant difference between winter and summer at individual time points from Sidak *post hoc* testing. Lower‐leg passive heating was analysed by two‐way repeated‐measures ANOVA.

The absolute value of *T*_re_ at baseline was higher in summer compared with winter in Fan trials (*P =* 0.001). Moreover, *T*_re_ was higher in the summer compared with the winter across the entire passive heating duration [average value: 37.31 (0.23)°C vs. 37.11 (0.28)°C, *P =* 0.0433; Figure 3a] in Fan trials. Mean *T*_sk_ at baseline was higher in the summer compared with winter [32.87 (0.62)°C vs. 33.49 (0.46)°C, *P =* 0.00312]. Mean *T*_sk_ did not differ between the two seasons in the Fan trials during the whole passive heating [34.75 (0.59)°C vs. 34.82 (0.45)°C, *P =* 0.795]. The absolute value of *T*_b_ at baseline was higher in summer compared with winter in Fan trials (*P =* 0.0410). Furthermore, *T*_b_ did not differ between the two seasons in the Fan trials [34.64 (0.26)°C vs. 34.86 (0.27)°C, *P =* 0.0631], and we did not observe a seasonal difference in all the time points from *post hoc* testing (all *P* > 0.237; Figure 3e).

#### 3.3.3 Thermoeffector and cardiovascular response

**FIGURE 4:**
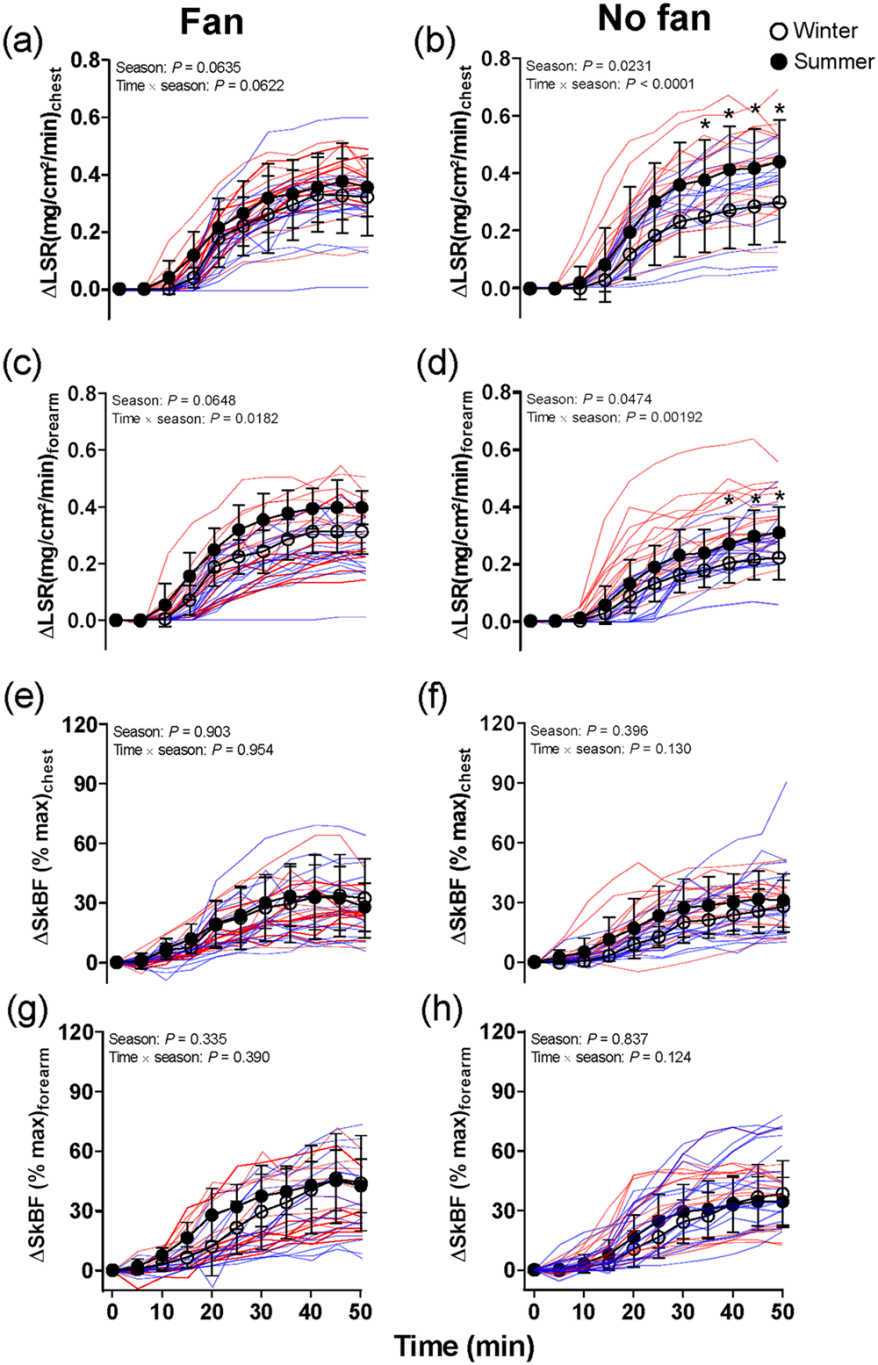
Time courses of change in local sweat rate (ΔLSR) on the forearm and chest (a–d) and skin blood flow (ΔSkBF) on the forearm (g, h) (*n* = 16; 8 males and 8 females) and the chest (e, f) (*n *= 15, 7 males and 8 females) during lower‐leg immersion between winter and summer in Fan and No fan trials. Filled circles are group means (SD) in summer, and open circles indicate group means (SD) in winter. The blue lines are individual responses in winter, and the red lines indicate the individual responses in summer. ^*^Significant difference between winter and summer at individual time points from Sidak *post hoc* testing. Lower‐leg passive heating was analysed by two‐way repeated‐measures ANOVA.

The ΔLSR at the chest during passive heating was not different between the two seasons in the Fan trials [0.16 (0.13) vs. 0.21 (0.15)) mg/cm^2^/min, *P =* 0.0635; Figure 4a]. We also did not observe a seasonal difference of ΔLSR in the forearm [0.14 (0.11) vs. 0.19 (0.13) mg/cm^2^/min, *P =* 0.0648; Figure 4c]. Furthermore, WBSR during passive heating was higher in the summer compared with the winter in the Fan trials [0.38 (0.13) vs. 0.28 (0.09) L/h, *P =* 0.00139, *d* = 0.93].

When expressing LSR_chest_ and LSR_forearm_ relative to *∆T*_b_, we observed that the *∆T*_b_ at onset of sweating was not different between the two seasons in Fan trials (all *P >* 0.0833; Table 4). Furthermore, the slope was greater in the summer compared with the winter in the Fan trials (all *P <* 0.0213) at the chest and forearm.

**TABLE 4:** The onset mean body temperature for local sweat rate and sensitivity of the relationships between mean body temperature and local sweat rate at each site of the body in two trials with (Fan) and without the electric fan (No fan) following seasonal acclimatization (*n* = 16).

	Fan				No fan			
Parameter	Mean ± SD		*P*‐value	Effect size, *d*	Mean ± SD		*P*‐value	Effect size, *d*
	Winter	Summer	Seasonal	Seasonal	Winter	Summer	Seasonal	Seasonal
Onset *T*_b_ (°C)								
Chest	0.63 ± 0.15	0.54 ± 0.07	0.0833	0.52	0.66 ± 0.09	0.56 ± 0.09	0.0147^∗^	0.98
Forearm	0.62 ± 0.08	0.53 ± 0.19	0.0894	0.52	0.64 ± 0.12	0.56 ± 0.11	0.0198^∗^	0.97
Slope (mg/cm^2^/min/°C)								
Chest	0.76 ± 0.31	0.97 ± 0.46	0.0213^∗^	0.64	0.87 ± 0.41	1.16 ± 0.55	<0.0001^∗^	0.88
Forearm	0.57 ± 0.35	0.82 ± 0.46	0.0139^∗^	0.69	0.71 ± 0.33	0.92 ± 0.39	0.0183^∗^	0.81

^*^Significant difference between winter and summer seasons (*P*‐values are reported).

Absolute heart rate at baseline was not different [66 (7.2) versus 65 (5.6) beats/min, *P =* 0.753] between the two seasons in the Fan trials. There was also no main effect for the season and no significant difference at all the time points in *post hoc* testing (all *P *> 0.198).

During lower‐leg immersion, ∆SkBF%_max_ at the chest and forearm showed no main effect of season and interaction effect (time × season; all *P >* 0.337; Figure 4e, g).

When expressing the SkBF_chest_ and SkBF_forearm_ relative to *∆T*_b_, the *∆T*_b_ onset of cutaneous vasodilatation occurred at a lower *∆T*_b_ in the summer compared with the winter season in Fan trials [0.63 (0.09)%max vs. 0.52 (0.17)%max on chest, 0.62 (0.12)%max vs. 0.52 (0.21)%max, all *P <* 0.0271].

#### 3.3.4 Thermal perceptions

**FIGURE 5:**
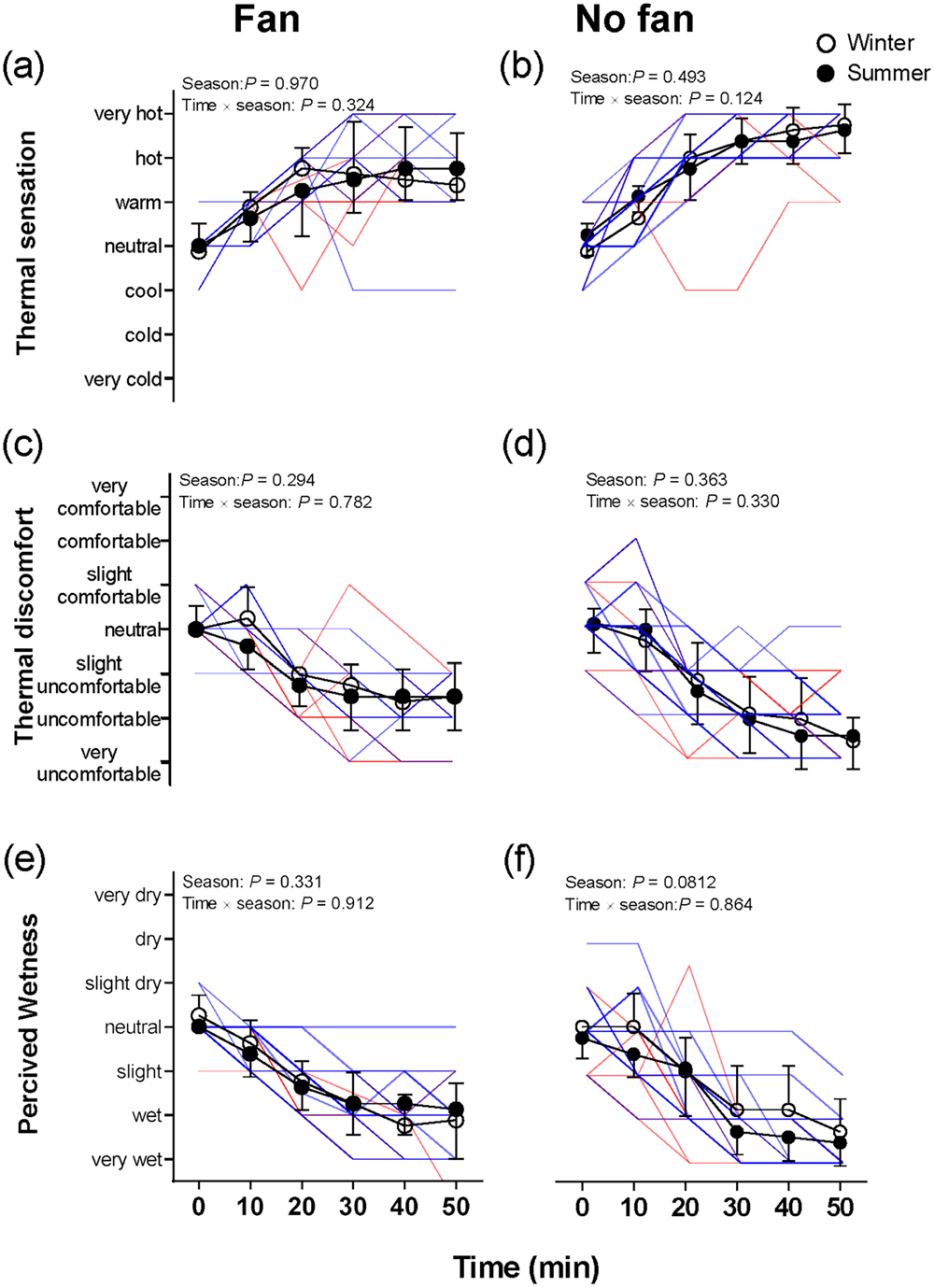
Time courses of thermal sensation (a, b), thermal discomfort (c, d) and wetness (e, f) on the whole body during lower‐leg immersion between No fan and Fan trials in winter and summer. *n* = 16 (8 males and 8 females). Filled circles are group means (SD) in summer, and open circles indicate group means (SD) in winter. The blue lines are individual responses in winter, and the red lines indicate the individual responses in summer; the individual lines might not be distinctly visible in some areas owing to overlapping. ^*^Significant difference between winter and summer at individual time points from Sidak *post hoc* testing. Lower‐leg passive heating was analysed by two‐way repeated‐measures ANOVA.

Thermal sensation, thermal discomfort and wetness perception at baseline were not different between the winter and summer in Fan trials (all *P >* 0.0830; Figure 5a,c,e). When comparing seasonal differences for thermal sensation and thermal discomfort, there were no significant differences between seasons in the Fan trials across the entirety of passive heating (all *P >* 0.294; Figure 5a,c). Specifically, no interactional effect (season × time) was observed in Fan trials (all *P >* 0.324). Moreover, wetness also was not different between the two seasons in Fan trials (*P = *0.331; Figure 5e).

### 3.4 Effect of seasonal heat acclimatization on perceptual and autonomic responses during passive heat stress

#### 3.4.1 Body temperature

The absolute value of *T*_re_ at baseline was higher in summer compared with winter in No fan trials (*P =* 0.0133). Moreover, *T*_re_ was higher in the summer compared with the winter across the entire passive heating duration (average value: 37.29°C vs. 37.06°C in No fan trials, *P =* 0.0328; Figure 3b) in No fan trials.

Mean *T*_sk_ at baseline was higher in the summer compared with the winter (32.96°C vs. 33.63°C, *P =* 0.00218). Mean *T*_sk_ was significantly higher in the summer compared with the winter in the No fan trials (34.79°C vs. 35.40°C, *P <* 0.0001; Figure 3d). The *T*_b_ was significantly higher in summer compared with winter in the No fan trials (36.61°C vs. 36.91°C, *P =* 0.0137; Figure 3f), but we did not observe a seasonal difference in all the time points from *post hoc* testing (all *P* > 0.310; Figure 3f).

#### 3.4.2 Thermoeffector and cardiovascular response

The ΔLSR at the chest during passive heating was significantly higher in summer compared with winter in No fan trials [0.16 (0.13) vs. 0.23 (0.17) mg/cm^2^/min, *P =* 0.0231; Figure 4b). The same differences were also observed in the forearm [No fan: 0.15 (0.12) vs. 0.21 [0.14] mg/cm^2^/min, *P =* 0.0474; Figure 4d]. Furthermore, we also observed that WBSR during passive heating was higher in the summer compared with the winter in No fan trials [0.41 (0.14) vs. 0.30 (0.09) L/h, all *P <* 0.0001, *d *= 0.87].

When expressing LSR_chest_ and LSR_forearm_ relative to ∆*T*_b_, we observed that the onset of sweating occurred at a lower ∆*T*_b_ in the summer compared with the winter in No fan trials (all *P <* 0.0147; Table 4). Furthermore, the slope was higher in the summer compared with the winter in the No fan trials (all *P <* 0.0183) at the chest and forearm.

Absolute heart rate at baseline was not different between the two seasons [68 (8.2) vs. 67 (6.2) beats/min, all *P >* 0.757]. Moreover, there was an interaction effect (time × season, all *P =* 0.001), with no main effect for the season and significant difference from *post hoc* testing (all *P >* 0.235).

During lower‐leg immersion, ∆SkBF%_max_ at the chest and forearm showed no main effect of season, similar between two seasons (all *P >* 0.396; Figure 4f,h). When expressing the SkBF_chest_ and SkBF_forearm_ relative to ∆*T*_b_, the onset of cutaneous vasodilatation was similar between seasons in No fan trials [0.59 (0.15)%max vs. 0.56 (0.23)%max on chest, 0.61 (0.27)%max vs. 0.57 (0.34)%max on the forearm, all *P >* 0.591].

#### 3.4.3 Thermal perceptions

Thermal sensation, thermal discomfort and wetness perception at baseline were not different between the winter and summer in the No fan trials (all *P >* 0.339). When comparing seasonal differences for thermal sensation, thermal discomfort and wetness, there were no significant differences between seasons in the No fan trials across the entirety of passive heating (all *P >* 0.0812; Figure 5b,d,f). Specifically, no interactional effect (season × time) was observed in the No fan trials (all *P >* 0.124).

## 4 DISCUSSION

The new finding that we identified from this study is that seasonal heat acclimatization enhanced the cool‐seeking behaviour; specifically, cool‐seeking behaviour (the electric fan use) was initiated earlier, more frequently and at a lower *∆T*_re_, ∆*T*_sk_ and *∆T*_b_ in the summer compared with the winter during passive heat stress.

### 4.1 The effect of seasonal heat acclimatization on cool‐seeking behaviour

We have observed that seasonal heat acclimatization affects cool‐seeking behaviour during passive heat stress. This is best evidenced by the observation that voluntary electric fan use was initiated at a lower ∆*T*_re_, *∆T*_sk_ and ∆*T*_b_, with total cooling time being longer in summer compared with winter (Table 3). The candidate mechanism to explain the early initiation of cool‐seeking behaviour is probably attributable to the higher *T*_re_, *T*_sk_ and *T*_b_ at baseline. The first cool‐seeking behaviour was initiated at the same thermal input, such that there was no difference in *T*_re_, *T*_sk_ and *T*_b_ between the two seasons when the fan was first turned on (Table 3). These results indicate that the absolute thermal inputs for activating cool‐seeking thermal behaviour were not altered by seasonal heat acclimatization. The total cooling time and cumulative times of turning on the fan after the initiation of the first behaviour were higher in the summer compared with the winter (Table 3). The thermal behavioural adaptations we observed might be attributable to the higher *T*_re_ observed in the summer during the passive heating (Figure 3). It has been reported that sweating responses to changing thermal inputs were improved by seasonal heat acclimatization (Lei et al., 2021; Nakamura & Okamura, 1998). Therefore, we also tried to evaluate the response of thermal behaviour with increasing *T*_b_ and found that the slope of the relationship between cumulative times of pressing the button and rising *T*_b_ was higher in summer than in winter. It has been shown previously that muscarinic receptors are more sensitive to thermal information in summer (Nakamura & Okamura, 1998; Ogawa & Sugenoya, 1993; Shin et al., 2016), and increased dopamine/decreased serotonin in the preoptic area and frontal cortex have been observed after repeated heat exposure (Clark & Lipton, 1985; Nakagawa et al., 2016). These previous findings support the possibility that repeated heat exposure and elevated *T*_re_ can induce higher responsiveness of the behavioural thermoregulation to thermal inputs in summer.

It has been reported that skin wetness, rather than skin wetness perception, is an important input for the initiation of cool‐seeking behaviour during passive heating (Vargas et al., 2020). In this study, WBSR was higher in summer compared with winter, although we did not observe a seasonal difference in wetness perception in this study. Also, we did not find significant differences in perceptions of Fan condition between summer and winter (Figure 5). Thus, these results suggest that the physiological skin wetness (e.g., LSR and WBSR) rather than skin wetness perception contributed to cool‐seeking behaviour in the model used in this study, which was also supported by a previous study reporting that physiological skin wetness is the primary mechanism contributing to thermal behaviour (Vargas et al., 2020). Wetness perception is a combination of tactile (i.e., stickiness) and thermal (i.e., warm) cues (Filingeri et al., 2015). It is possible that the mechanoreceptor (i.e., LSR, WBSR) alone could bypass the medial prefrontal cortex where the thermal behaviour decision was made. Future studies investigating the control of cool‐seeking behaviour in humans should measure skin wetness.

Interestingly, we also observed that cool‐seeking behaviour was initiated before autonomic thermoeffectors (SR and SkBF) in both seasons, which indicated that the order of thermoregulatory responses to heat stress is not modified by seasonal heat acclimatization, because both autonomic and behavioural thermoregulation were adapted to heat stress after seasonal heat acclimatization. It was supported by previous studies that the thermoeffectors are recruited in an orderly manner, and thermal behaviour was initiated before autonomic thermoeffectors (SR and SkBF), seemingly to save physiological energy (Schlader & Vargas, 2019; Schlader et al., 2018).

### 4.2 The effect of seasonal heat acclimatization on thermal perception

We did not observe a difference in thermal perception in the Fan and No fan trials between summer and winter. Sotiridis et al. (2020) showed that participants responded at the same comfort temperature zone during passive heating before and after heat acclimation, which is consistent with our results. In contrast, Lee et al. (2011) demonstrated that tropical indigenes detected warm and hot sensations at higher *T*_sk_ than the indigenes of temperate climates in all body regions, after long‐term heat acclimatization. Thus, it is suggested that seasonal heat acclimatization in the present study and short‐term heat acclimation (Sotiridis et al., 2020) do not affect thermal perceptions during passive heating. Moreover, the different durations of heat exposure and methods might be related to our conflicting results (Lee et al., 2011), indicating that the temporal changes in thermal perception during passive heating associated with heat acclimation and acclimatization require further investigation.

### 4.3 The effect of seasonal heat acclimatization on autonomic thermoeffectors

The effect of seasonal heat acclimatization on autonomic thermoeffectors that we observed in No fan trials (e.g., *T*_sk_, *T*_b_, LSR was higher, and the onset of LSR was earlier; Table 4) disappeared when behaviour was allowed in Fan trials. Those findings are in agreement with our hypothesis.

There was an observed enhancement of autonomic thermoeffector responses in the No fan trials, such as higher *T*_sk_, *T*_b_ and LSR (Figures 3 and 4), and the onset was earlier, consistent with previous studies (Hori et al., 1974; Inoue et al., 1995; Lei et al., 2021). This enhancement could be attributed to the increased reactivity of muscarinic receptors to cholinergic stimuli during the summer (Banjar et al., 1989; Shibasaki et al., 2006; Shin et al., 2016). Supporting this explanation, our results showed higher LSR at the forearm and increased responses in the pilocarpine–iontophoresis test following seasonal heat acclimatization. Furthermore, the higher *T*_re_, *T*_sk_ and *T*_b_ at the baseline that we observed in this study, which might have been attributable to a higher ambient temperature in summer, were necessary to maintain the heat dissipation from the skin surface to the surroundings and heat transfer from the core to the skin surface.

However, unlike the No fan trials, we did not observe this seasonal difference in *T*_b_, *T*_sk_, LSR and onset of LSR in the Fan trial (all *P >* 0.0631), which is probably attributable to cool‐seeking behaviour being initiated earlier and cooling time being longer in summer (Table 3). These results indicate that the augmented response of thermoeffectors induced by seasonal heat acclimatization could be attenuated using cool‐seeking behaviour in summer.

### 4.4 Considerations and limitations

First, we acknowledge that we did not record the total outdoor exposure time of our participants in both seasons and, therefore, there might be different levels of adaptations across different participants, and the results might differ in other countries (e.g., see our ambient temperature and humidity). However, this would not affect our primary markers, such as LSR and WBSR, because our thermoeffector responses demonstrated a typical pattern of heat acclimatization, such as the increase of sweating from iontophoresis. We did not record the physical activity of participants, hence we could not fully explain the decreased ${\dot{V}}_{O_{2}\mathrm{peak}}$ in summer in the present study. However, it was reported that physical activity enhances sweating in young adults (Amano et al., 2017; Kuwahara et al., 2005). Therefore, it might be that the seasonal difference we observed in the present study would be bigger if we matched the ${\dot{V}}_{O_{2}\mathrm{peak}}$ between the seasons by controlling the activity of subjects. We also did not randomize the order of Fan and No fan trials, which might have resulted in bias.

Second, the thermal perception measurement scales used in this study were seven‐point scales from the study by Olesen & Brager (2004), which might not have been sufficiently sensitive to identify small changes in thermal perception. Furthermore, the air velocity we used in the present study was designed to be suitable for use in daily life at rest in the winter and summer months. However, further investigation is required to gain a better understanding of the effect of air velocity on behavioural thermoregulation during heat stress.

Finally, because the airflow of the fan we used in this study is 0.71 (0.07) m/s in winter and 0.72 (0.06) m/s in summer, the effect of cooling behaviour (i.e., fan use) on autonomic thermoeffectors might not have been sufficient in the present study. However, it remains notable that the cooling behaviour was adequate for the participants to be willing to turn on the fan. As elucidated in the antecedent discourse, the disparity in the mean onset time of the initial cool‐seeking behaviour between the two seasons amounts to <5 min. Nonetheless, given our thermal perception executed at 10 min intervals, this raises a pronounced probability of overlooking potential differences owing to the wide temporal span for our perceptual measures.

### 4.5 Perspectives and significance

The findings from the present study highlight that cool‐seeking behaviour (i.e., voluntary electric fan use) during passive heat stress differs between winter and summer months, such that cool‐seeking behaviour was engaged to a greater extent to achieve similar levels of thermal comfort and sensation in the summer. Seasonal autonomic adaptation might be compensated for by the enhanced behaviour, and this could help people to use behaviour to avoid physiological strain. Meanwhile, it also indicated that the reliance on behaviours often consumes more energy and is less sustainable. This knowledge can inform the development of climate adaptation strategies for individuals and communities. It also can inform workplace safety guidelines and/or enhance safety during activities in the heat.

## 5 CONCLUSION

We have demonstrated that seasonal heat acclimatization influences both autonomic and behavioural thermoregulation. Specifically, cool‐seeking behaviour was initiated at lower increases in *T*_re_, *T*_sk_ and *T*_b_ and it was used to a greater extent in summer compared with winter. However, thermal perception was not modified by seasonal heat acclimatization in this study.

## AUTHOR CONTRIBUTIONS

Hui Wang, Zachary J. Schlader, Tze‐Huan Lei and Narihiko Kondo contributed to conceptualization and design. Hui Wang and Narihiko Kondo were responsible for data collection and data analysis. Hui Wang, Narihiko Kondo and Zachary J. Schlader were responsible for the interpretation and drafting of the article. Zachary J. Schlader, Toby Mündel, Tatsuro Amano, Naoto Fujii and Takeshi Nishiyasu reviewed the article and provided critical feedback. All authors approved the final version of the manuscript and agree to be accountable for all aspects of the work in ensuring that questions related to the accuracy or integrity of any part of the work are appropriately investigated and resolved. All persons designated as authors qualify for authorship, and all those who qualify for authorship are listed.

## CONFLICT OF INTEREST

The authors declare no conflicts of interest.

## Supporting information



Supplementary Materials

Supplementary Materials

Supplementary Materials

## Data Availability

Data will not be shared in public and will be available upon request by the readers with the contact of the corresponding author.
